# OA09.03. Stress reduction using massage in cardiac surgery patients

**DOI:** 10.1186/1472-6882-12-S1-O35

**Published:** 2012-06-12

**Authors:** L Braun, C Stanguts, L Casanelia, O Spitzer, E Paul, N Vardaxis, F Rosenfeldt

**Affiliations:** 1 Monash, Melbourne, Australia; 2 Endeavour College, Melbourne, Australia

## Purpose

The primary aim of the study was to determine the effects of massage therapy, delivered to post-surgery cardiothoracic patients, on pain, anxiety, relaxation and muscular tension at two different time points and compare it to an equivalent period of rest time. The secondary aims were to explore the effects of massage on heart rate, respiratory rate and blood pressure, feasibility of treatment delivery in a busy ward and staff acceptance of the new therapy and therapists.

## Methods

A randomised study conducted at the Alfred hospital compared massage therapy to an equivalent period of rest time. Visual analogue scales measured pain, anxiety, relaxation, muscular tension and satisfaction. Heart rate, respiratory rate and blood pressure were measured pre- and post-treatment by a cardiac nurse. Focus groups, staff and therapist feedback were utilised to collect qualitative data about clinical significance and feasibility of delivering the treatment.

## Results

One hundred and fifty-two (99% response rate) patients participated. Compared to rest time, massage therapy produced a significantly greater reduction in pain (p=0.001), anxiety (p<0.0001), muscular tension (p=0.002) and increases in relaxation (p<0.0001) and satisfaction (p=0.016). No significant differences were seen for heart rate, respiratory rate and blood pressure. The effects at different time points were also compared between the groups. Pain significantly reduced after massage on day 3/4 (p<0.0001) and day 5/6 (p=0.003) whereas controls experienced no significant change at either time point. At both time points, massage significantly reduced anxiety (p<0.0001) and muscular tension (p<0.0001). Relaxation significantly improved on day 3/4 for both groups (massage p<0.0001; rest time p=0.006) but only massage was effective on day 5/6 (p<0.0001). Nurses and physiotherapists confirmed patient improvements and helped facilitate delivery of the treatment.

## Conclusion

Post-surgery massage therapy significantly improved pain, anxiety, muscular tension and relaxation amongst cardiothoracic surgery patients, was well accepted and feasible to deliver.

